# The challenge of breath: toward an ‘after’ COVID‐19

**DOI:** 10.1111/1469-8676.12806

**Published:** 2020-05-20

**Authors:** Marsha Rosengarten

**Affiliations:** ^1^ Department of Sociology, Goldsmiths University of London New Cross London SE14 6NW UK

COVID‐19 has the capacity to take our breath away, the very essence of what is necessary to sustain life. So too might the response to COVID‐19 be seen as breathtaking. Besides the introduction, almost overnight, of extraordinary changes to our daily existence – notably practices of isolation, paradoxically consistent with the necessity for collectively thwarting COVID‐19’s communicability – science is also altering apace. New collaborations are rapidly arising across a globally dispersed bioscientific field, aimed at devising and testing innovative therapeutics and vaccines.

It would be easy to attribute these myriad changes to the virility of the virus itself and, also, assume science will be our saviour. To be sure, science has made a significant difference to viral infections such as HIV and Ebola. Indeed, we might well leave science to its work on the virus and, instead, as some argue, focus critical attention on modes of neoliberalism and structures of corporate capitalism that can be held responsible for the impoverishment that contagious infections feed on.

But as much as science might be our ally, it is not immune to question. The first principle of science is that existence is composed of independent physical objects and events, of ‘things’ essentially isolated and that interact with finite consequences. Each event makes its difference in a linear succession. Perhaps with a residue, but as if without immanent connection and creativity for what becomes. Breath in this schema is imagined as essential but, nonetheless, no more than an exchange of elements between isolated beings, elements of plant and animate life (including viral agents) and what they are thought to ‘contain’ or unleash.

Yet, the possibility for breath, its ‘communicability’ and no less COVID‐19 suggests – as A.N. Whitehead and other process thinkers would propose – that any scientific formulation is a partial understanding, a partiality from more. The formulation may provide sufficient for devising interventions that can be demonstrated to save lives. But this should not be mistaken for an efficacy of thought that proceeds by excluding and, thus, compartmentalising the essential connectedness that enables breath and, simultaneously, creates for what matters of life.

If we are to contemplate an ‘after’ to COVID‐19, including the anticipation of a future of more novel deadly infections, might breath’s essential connectiveness, its creative preciousness and, thus, also its precariousness offer an element (not a principle) for learning? Might this dimension of our collective connectedness to the world provide cause for a pause in the atomistic logic of scientific thought? A pause of the kind we experience when taking a breath to sustain our own creative existence and, with its taking, may appreciate the creativity that makes experience possible? To pursue this requires not the rejection of science, but rather a counter to relying on it without appreciating the cost to an immanent creativity by its logic. In sum, I offer a call to appreciate an ongoing creative process that might enable us to become more creative in our response.

